# Supplementary material for: Multi-input CRISPR/Cas genetic circuits that interface host regulatory networks
Source: Mol Syst Biol. 2014 Nov 24;10(11):763. doi: 10.15252/msb.20145735 (PMC4299604; doi:10.15252/msb.20145735)
Supplement: Supplementary file 1 — Supplementary Information [file msb0010-0763-sd1.pdf]

Supplemental Information for:

Multi-input CRISPR/Cas genetic circuits that interface host regulatory networks

Alec A. K. Nielsen & Christopher A. Voigt

I. Measurement of NOT gate response functions 2
II. Cytometry data for sgRNA orthogonality 4
III. Design of sgRNA sequences 5
IV. Comparison of response functions for sgRNAs and TetR-family repressors 6
V. Toxicity of sgRNA expression 7
VI. Cytometry data for genetic circuits 8
VII. Plasmid maps and part sequences 9
VIII. Supplementary References 14

I. Measurement of NOT gate response functions

The response function of a NOT gate captures how the output promoter changes as a function of the input promoter. Because the gate is measured using an inducible promoter (in our case arabinose-inducible P_{BAD}), the concentration of inducer has to be exchanged for the activity of the inducible promoter[1]. To do this, the activity of P_{BAD} is measured as a function of [arabinose] and this is used to rescale the input (x-axis of the function). For example, to generate the response function for sgRNA-1T (Figure 2e), we induced cells harboring pAN-P_{BAD}-sgRNA-A1T, pAN-P_{A1}-RFP, and pAN-P_{Tet}-dCas9 in 0.625 ng/mL aTc and various arabinose concentrations, and then performed flow cytometry (Figure S1, bottom panel). Additionally, in order to determine what the underlying activity of P_{BAD} was in these experiments, we induced cells harboring pAN-P_{BAD}-YFP in an identical manner (Figure S1, top panel).

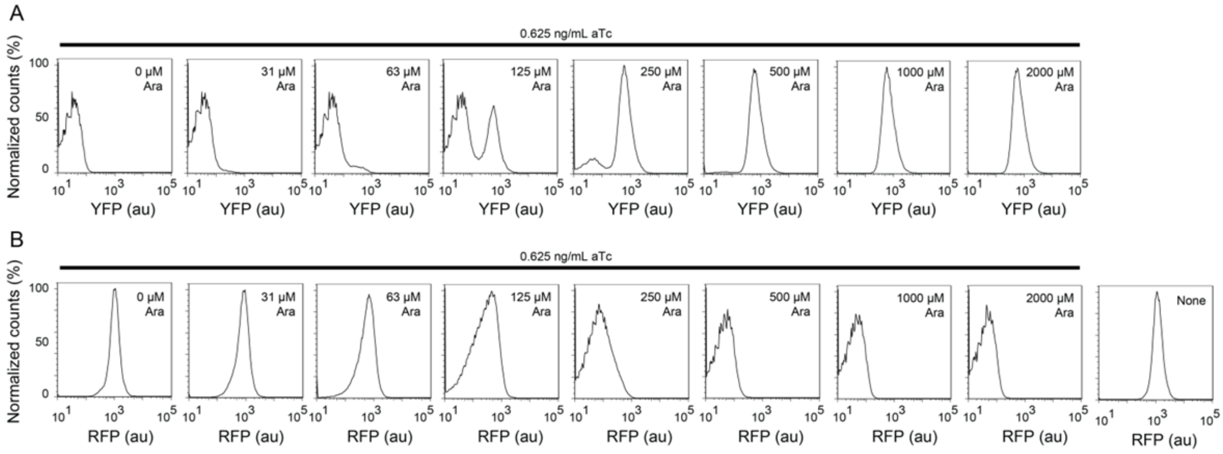

Figure S1: Cytometry data used to rescale the response function of the NOT gate based on sgRNA-A1T. (A) *YFP histograms for the inducible promoter control, P_{BAD} driving YFP (plasmids pAN-P_{Tet}-dCas9 and pAN-P_{A1}-RFP). All samples were grown in the presence of 0.625 ng/mL aTc to induce dCas9 and the stated amount of arabinose.* ***(B)*** *The raw data for the response function of the NOT gate based on sgRNA-A1T is shown. All samples were grown in the presence of 0.625 ng/mL aTc to induce dCas9 and the stated amount of arabinose, except for the right-most histogram which was grown in the absence of both inducers and provides a maximum achievable reporter output for P_{A1}-RFP. Plasmid maps are shown in Figure S8.*

A plot of P_{BAD}-YFP as a function of arabinose shows the plateaus of the promoter at low and high arabinose concentrations (Figure S2a). Similarly, a plot of P_{A1}-RFP as a function of arabinose shows a similar plateauing at high and low concentrations due to the underlying P_{BAD} saturation (Figure S2b). In order to visualize the relationship between P_{BAD} activity and P_{A1} activity, we convert the x-axis of Figure S2b to units of P_{BAD}-YFP (Figure S2c). The response functions for all sgRNAs with their cognate promoters are shown in Figure S3.

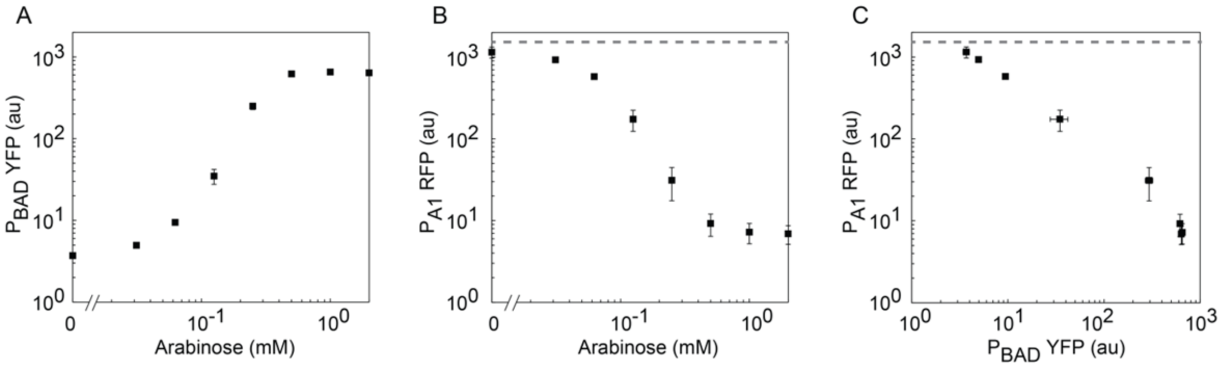

Figure S2**: Creation of the response function for the NOT gate (sgRNA-A1T). (A)* *The data shown is for the induction of P_{BAD} and is calculated using the geometric mean of the cytometry data in Figure S1 (top).* ***(B) *The activity of P_{A1}-RFP output as a function of arabinose. Dashed lines indicate the maximum achievable RFP output, determine from an experimental treatment where dCas9 was not induced (Figure S1 bottom, rightmost panel).* ***(C)*** *The x-axis of the P_{A1}-RFP plot is transformed to P_{BAD}-YFP units to visualize the relationship between the input promoter that drives sgRNA-A1T and the cognate repressible promoter that drives RFP. Data points represent the average and standard deviation of three experiments.*

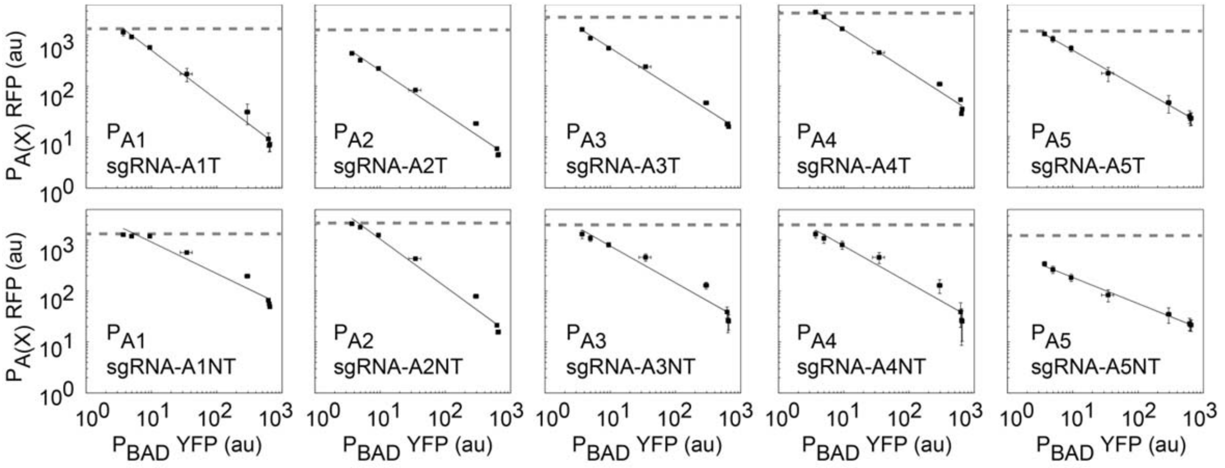

Figure S3: Response functions for all of the NOT gates based on orthogonal sgRNAs repressing their cognate promoters. *Dashed lines indicate the maximum achievable RFP output, determine from an experimental treatment where dCas9 was not induced. Solid lines are power law fits to the data and correspond to the lines shown in the Figure 2e inset. Data points represent the average of the geometric means of three experiments on different days.*

II. Cytometry data for sgRNA orthogonality

The programmability of RNA-DNA interactions potentially allows for a large number of orthogonal sgRNAs and cognate promoters to be designed. Figure S4 shows the raw data for the full orthogonality grid shown in Figure 2d. Although each template sgRNA shares its six 5'-nucleotides with every other template sgRNA in order to bind the -35 σ_{70}-binding site of the promoter (similarly for the non-template sgRNAs and the -10 σ_{70}-biding site), the subsequent twelve 3'-nucleotides are unique and comprise a "seed" region that does not tolerate mismatches.

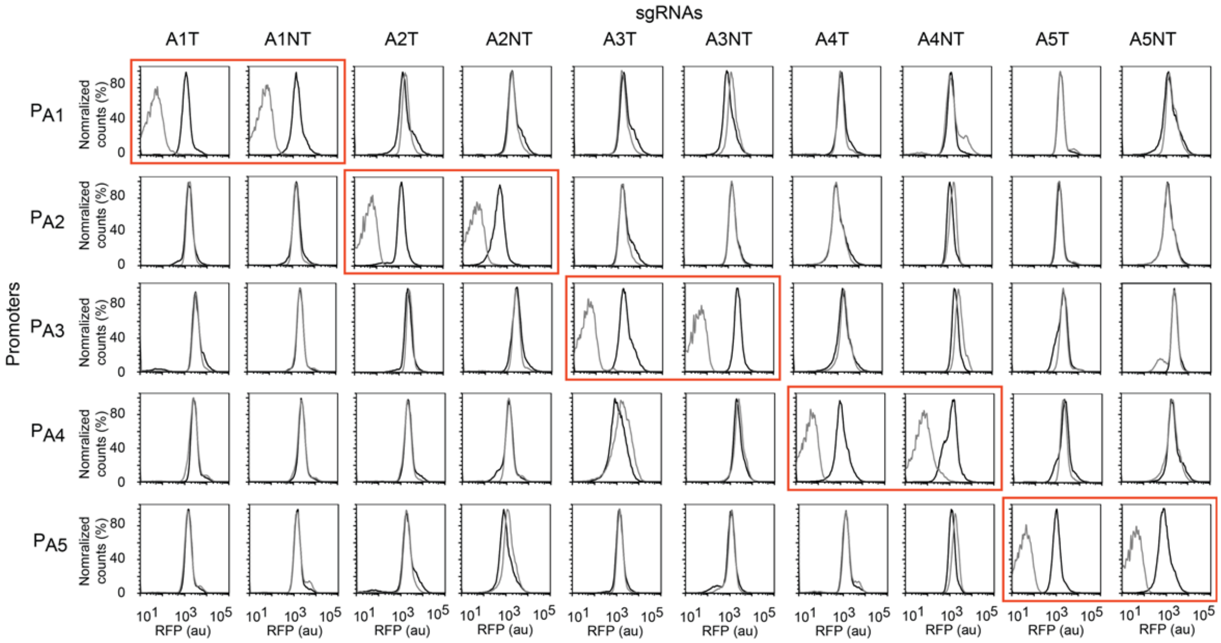

Figure S4: *Representative cytometry data corresponding to the values used for the cross-talk map in Figure 2d. Histograms are for RFP produced from pAN-$P_{A(X)}$-RFP while being repressed by sgRNAs produced from pAN-P_{BAD}-sgRNA-A(X)(T/NT). Cells also harbor pAN-P_{Tet}-dCas9. Black histograms correspond to dCas9 induction with aTc, but no sgRNA induction. Gray histograms correspond to dCas9 and sgRNA induction with 0.625 ng/mL aTc and 2mM arabinose, respectively. Red boxes indicate cognate sgRNA-promoter pairs.*

III. Design of sgRNA sequences

Each sgRNA was designed so that the first eight nucleotides of the guide region bind the -35 or -10 sites (for template and non-template targeting sgRNAs, respectively) followed by a 'CC' for the opposite strand's PAM. The subsequent twelve nucleotides of the guide region bind the promoter-specific sgRNA operator for each promoter P_{A1} through P_{A5}. Tables S1 and S2 list the sequences and fold-repression values for sgRNA NOT gates. Fold-repression values were calculated from the orthogonality grid experiment, and represent the RFP output for the uninduced state (no aTc, no arabinose) divided by the RFP output of the fully induced state (0.625 ng/mL aTc and 2 mM arabinose).

Table S1. Sequences and fold-repression values for template-targeting sgRNAs

Name	Region that binds -35 and **reverse PAM**	Region that binds the promoter-specific operator	Fold-repression
sgRNA-A1T	UUUACA**CC**	UAGCUCAGUCCU	280
sgRNA-A2T	UUUACA**CC**	AACGGGUCACAC	100
sgRNA-A3T	UUUACA**CC**	CGAAAUGGAGCA	220
sgRNA-A4T	UUUACA**CC**	UCCACAACUAGC	190
sgRNA-A5T	UUUACA**CC**	AAAACACUCGGA	440

Table S2. Sequences and fold-repression values for non-template-targeting sgRNAs

Name	Region that binds -10 and **forward PAM**	Region that binds the promoter-specific operator	Fold-repression
sgRNA-A1NT	AUAAUA**CC**	UAGGACUGAGCU	94
sgRNA-A2NT	AUAAUA**CC**	CGUGUGACCCGU	250
sgRNA-A3NT	AUAAUA**CC**	AUGCUCCAUUUC	340
sgRNA-A4NT	AUAAUA**CC**	AGCUAGUUGUGG	56
sgRNA-A5NT	AUAAUA**CC**	CUCCGAGUGUUU	270

IV. Comparison of response functions from gates based on sgRNA and TetR-family repressors

Figure S5 shows a comparison of response functions. Previously, we measured the response functions for a library of NOT gates based on TetR-family repressors. The average of 14 response functions is shown in Figure S5 (green line) along with the highest (LmrA) and lowest (BM3R1) individual response functions[2]. The average line was generated by calculating the average of a set of parameters (half-max threshold, Hill coefficient, maximum and minimum) and then generating a line corresponding to these parameters. The purple line is the power law fit to the sgRNA response function from Figure 3b. In Figure S5 (A), the average line, LmrA, and BM3R1 y-axis values are scaled by the maximum output value of the average line. Simiarly, the sgRNA output values are scaled by its maximum output value. The x-axis values are scaled by maximum values measured for the input promoter. In Figure S5 (B), both the y-axis and x-axis are scaled so that input and output range for both lines spans from 10^{-3} to 10^{0}. This is done to both show the difference in dynamic range (A) and overall shape (B) of the response functions.

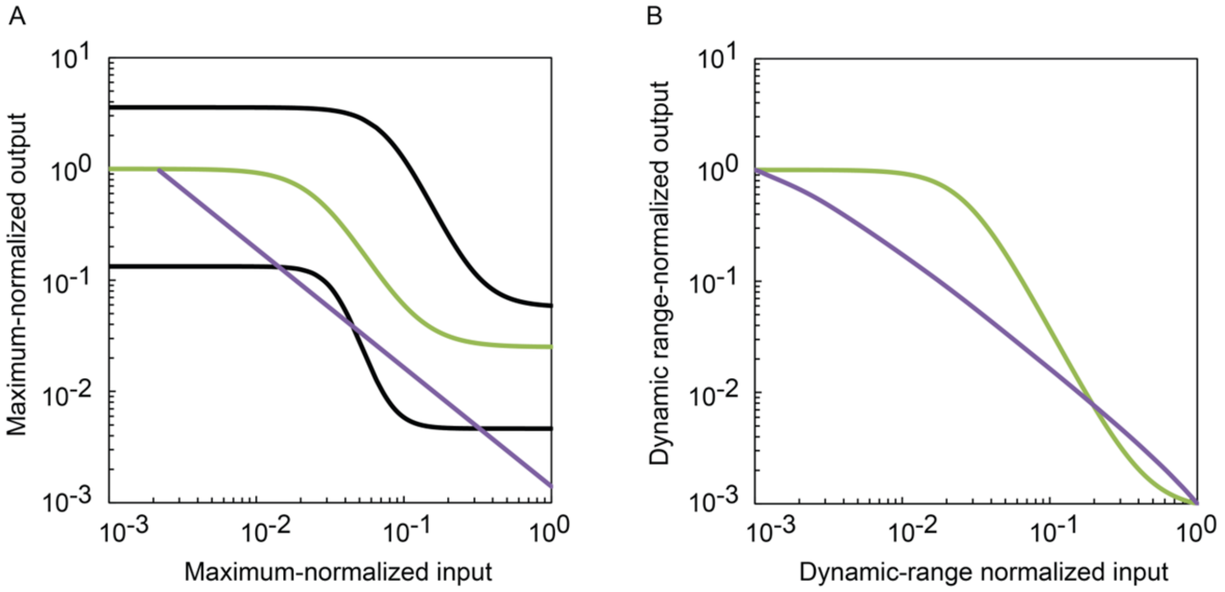

Figure S5: Comparison of NOT gate response functions generated by sgRNAs versus TetR-family repressors. *Purple lines: Power law fit to the sgRNA cascade relationship from Figure 3b. Green lines: Hill-function generated from the average Hill-equation parameters of 14 TetR homologues (K, n, max, min)[2]. Black lines: Hill-function fits to the highest and lowest response curves, LmrA and BM3R, respectively. (A) For dynamic range comparison, all input and output values are re-scaled so that their maxima equal 1 (except for the black line outputs, which are scaled using the green line maximum). (B) Same as in A, except the y-axis is also normalized by the minimum value to compare the shape of the curves.*

V. Toxicity of sgRNA expression

High expression of dCas9 can be very toxic to the host cell (Figure 2b). To determine the toxicity of sgRNA expression, we induced the expression of sgRNAs at various levels and measured the optical density after six hours. Both dCas9 and RFP were expressed in the cells as well. Two sgRNAs were tested: 1) an sgRNA that targets an operator in an otherwise functionless region of the high-copy sgRNA plasmid (blue squares), and 2) a scrambled sgRNA that does not target any DNA sequence in the cell (red squares). Only a slight decrease in the growth is observed and both the functional and scrambled sequence have identical behavior.

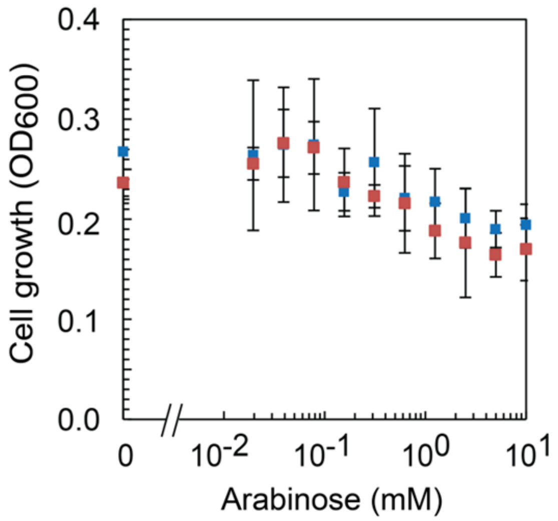

Figure **S6: Toxicity of sgRNA expression from an arabinose-inducible promoter.** Blue *squares: expression from pAN-P_{BAD}-sgRNA-VR, which binds an operator on its high-copy plasmid backbone. Red squares: expression from pAN-P_{BAD}-sgRNA-scramble, a “scrambled” sgRNA that does not target any genetic locus in the cell. All samples had dCas9 induced with 0.625 ng/mL aTc, and RFP constitutively expressed.*

VI. Cytometry data for genetic circuits

Representative fluorescence histograms corresponding to the five input states for the genetic circuits of Figure 3b (NOT-NOT), 3d (NOR), 3f (OR), 3h (AND) and Figure 4b (NOR from OR-MalT-3NT) are shown (Figure S7). Black histograms correspond to no induction of dCas9 and reflect the “maximum output” achievable. Colored histograms each have dCas9 induced and correspond to the four digital induction conditions for expressing input promoters.

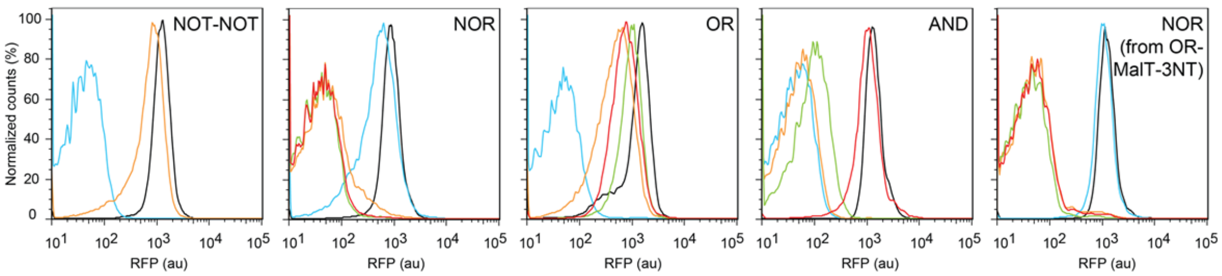

Figure S7: Histograms for genetic circuits encoded on pAN-NOT-NOT, pAN-NOR- pAN-OR, pAN-AND, and pAN-OR-MalT-3NT. *The black histograms indicate cultures without inducer and correspond to the maximum value achievable for the output promoters, blue is with 0.625 ng/mL aTc, orange is with 0.625 ng/mL aTc and 2mM arabinose, green is with 0.625 ng/mL aTc and 25 µM DAPG, and red is with 0.625 ng/mL aTc, 2mM arabinose, and 25 µM DAPG. The plasmid maps are shown in Figure S8 and S9.*

VII. Plasmid maps and part sequences

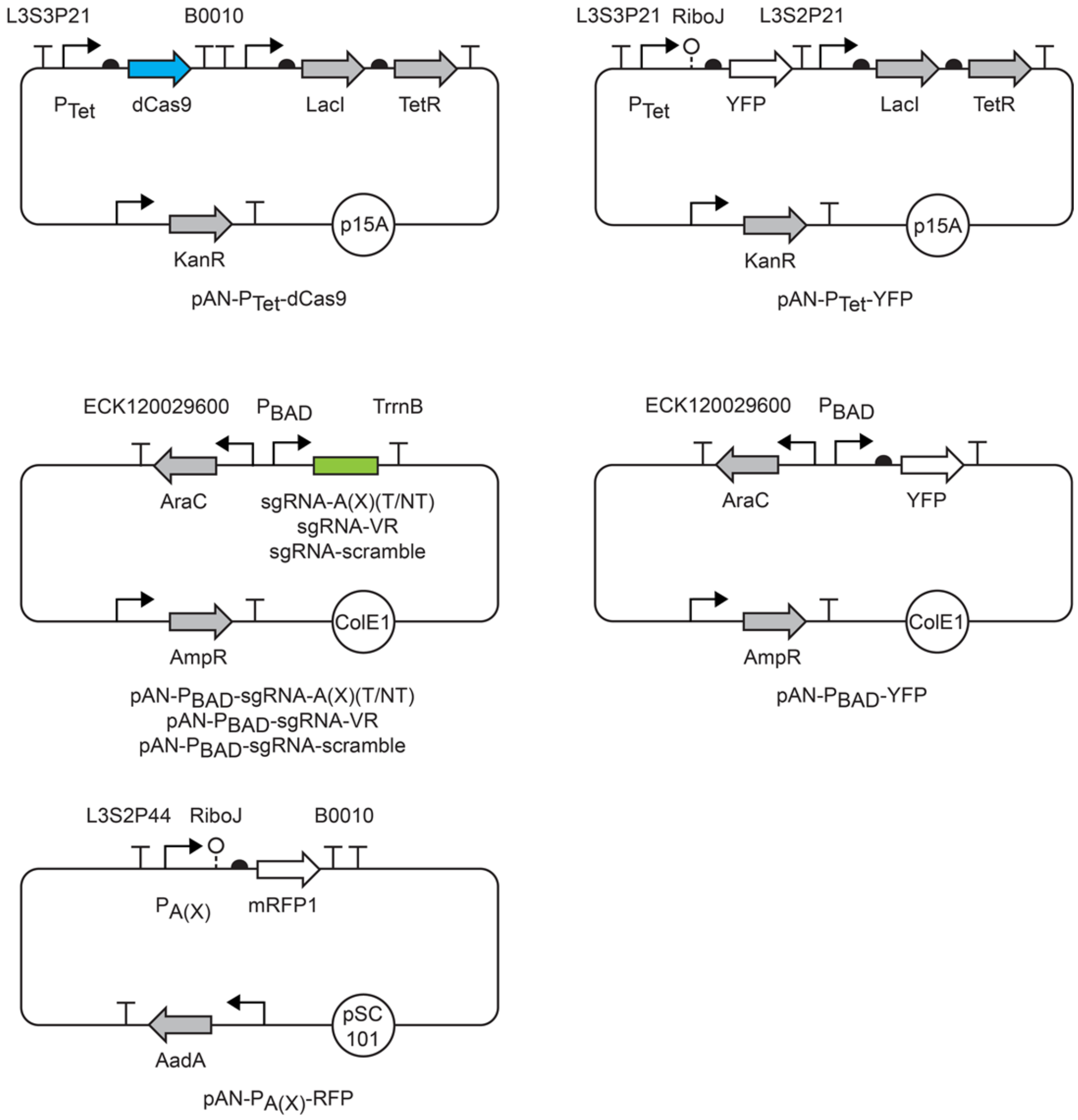

Figure S8: *Plasmids encoding basic circuit components. The pAN-P_{Tet}-dCas9 plasmid (p15A, KanR) encodes the insulated, tight-off aTc-inducible dCas9 used for all experiments. The pAN-$P_{A(X)}$-RFP series of reporter plasmids (pSC101, AadA) encode one of five synthetic sgRNA-repressible promoters that express mRFP1. The pAN-P_{BAD}-sgRNA-A(X)(T/NT) and pAN-P_{PhlF}-sgRNA-A(X)(T/NT) series of plasmids (ColE1, AmpR) drive one of ten sgRNAs from either the arabinose- or DAPG-inducible promoters, respectively. The pAN-P_{Tet}-YFP and pAN-P_{BAD}-YFP plasmids were used to characterize the promoter activities of P_{Tet} and P_{BAD}, respectively, for dCas9 and sgRNA response functions.*

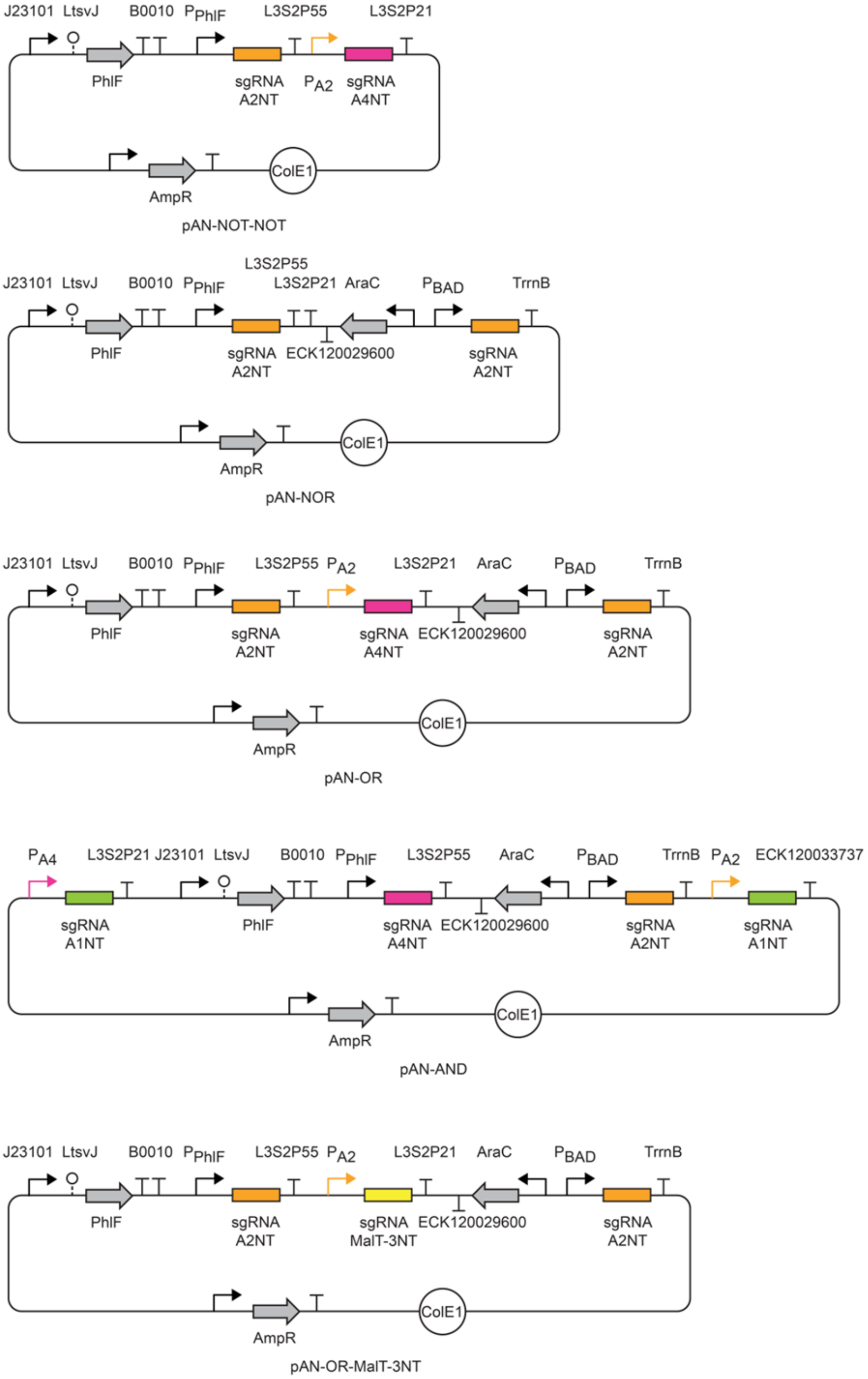

Figure S9: *Plasmids encoding genetic circuits. The pAN-NOT-NOT, pAN-NOR, pAN-OR, and pAN-AND plasmids encode the sgRNA circuits from Figure 3. The pAN-OR-MalT-3NT encodes the MalT knockdown logic circuit from Figure 4. These plasmids were co-transformed with pAN-P_{Tet}-dCas9 and the appropriate pAN-$P_{A(X)}$-RFP plasmids to implement complete systems.*

Table S3. Sequences of genetic parts used in this work

Part name	Type	DNA sequence
BBa_J23101	promoter	tttacagctagctcagtcctaggtattatgctagc
P_{Const}	promoter	gcggcgcgccatcgaatggcgcaaaacctttcgcggtatggcatgatagcgcccggaagagagtcaattcagggtggtgaat
P_{BAD}	promoter[3]	acttttcatactcccgccattcagagaagaaaccaattgtccatattgcatcagacattgccgtcactgcgtcttttactggctcttctcgctaaccaaaccggtaaccccgcttattaaaagcattctgtaacaaagcgggaccaaagccatgacaaaaacgcgtaacaaaagtgtctataatcacggcagaaaagtccacattgattatttgcacggcgtcacactttgctatgccatagcatttttatccataagattagcggatcctacctgacgctttttatcgcaactctctactgtttctccata
P_{PhlF}	promoter[2]	cgacgtacggtggaatctgattcgttaccaattgacatgatacgaaacgtaccgtatcgttaaggt
P_{Tet}	promoter[2]	tactccaccgttggctttttttccctatcagtgatagagattgacatccctatcagtgatagagataatgagcac
P_{A1}	promoter	tttacacctagctcagtcctaggtattatgctagc
P_{A2}	promoter	tttacaccaacgggtcacacgggtattatgctagc
P_{A3}	promoter	tttacacccgaaatggagcatggtattatgctagc
P_{A4}	promoter	tttacacctccacaactagctggtattatgctagc
P_{A5}	promoter	tttacaccaaaacactcggagggtattatgctagc
P_{AN} spacer	spacer	tccgaatgacatgcgtctccgttttagagctagaaatagcaagttaaaataaggctagtccgattgcaacttgaaaaagtggcaccgagtcggtgcttttttt
sgRNA-A1T	sgRNA	tttacacctagctcagtcctgttttagagctagaaatagcaagttaaaataaggctagtccgattgcaacttgaaaaagtggcaccgagtcggtgcttttttt
sgRNA-A1NT	sgRNA	ataatacctaggactgagctgttttagagctagaaatagcaagttaaaataaggctagtccgattgcaacttgaaaaagtggcaccgagtcggtgcttttttt
sgRNA-A2T	sgRNA	tttacaccaacgggtcacacgttttagagctagaaatagcaagttaaaataaggctagtccgattgcaacttgaaaaagtggcaccgagtcggtgcttttttt
sgRNA-A2NT	sgRNA	ataatacccgtgtgacccgtgttttagagctagaaatagcaagttaaaataaggctagtccgttatcaacttgaaaaagtggcaccgagtcggtgcttttttt
sgRNA-A3T	sgRNA	Tttacacccgaaatggagcagttttagagctagaaatagcaagttaaaataaggctagtccgattgcaacttgaaaaagtggcaccgagtcggtgcttttttt
sgRNA-A3NT	sgRNA	ataataccatgctccatttcgttttagagctagaaatagcaagttaaaataaggctagtccgattgcaacttgaaaaagtggcaccgagtcggtgcttttttt
sgRNA-A4T	sgRNA	tttacacctccacaactagcgttttagagctagaaatagcaagttaaaataaggctagtccgattgcaacttgaaaaagtggcaccgagtcggtgcttttttt
sgRNA-A4NT	sgRNA	ataataccagctagttgtgggttttagagctagaaatagcaagttaaaataaggctagtccgttatcaacttgaaaaagtggcaccgagtcggtgcttttttt
sgRNA-A5T	sgRNA	tttacaccaaaacactcggagttttagagctagaaatagcaagttaaaataaggctagtccgattgcaacttgaaaaagtggcaccgagtcggtgcttttttt
sgRNA-A5NT	sgRNA	ataataccctccgagtgtttgttttagagctagaaatagcaagttaaaataaggctagtccgattgcaacttgaaaaagtggcaccgagtcggtgcttttttt
sgRNA-VR	sgRNA	tgcgctcggtcgttcggctggttttagagctagaaatagcaagttaaaataaggctagtccgttatcaacttgaaaaagtggcaccgagtcggtgcttttttt
sgRNA-scramble	sgRNA	aacccctgattgtatccgcagttttagagctagaaatagcaagttaaaataaggctagtccgttatcaacttgaaaaagtggcaccgagtcggtgcttttttt
RiboJ	insulator[4]	agctgtcaccggatgtgctttccggtctgatgagtccgtgaggacgaaacagcctctacaaataattttgtttaa
LtsvJ	insulator[4]	agtacgtctgagcgtgatacccgctcactgaagatggcccggtagggccgaaacgtacctctacaaataattttgtttaa
dCas9	gene[5,6]	atggataagaaatactcaataggcttagctatcggcacaaatagcgtcggatgggcggtgatcactgatgaatataaggttccgtctaaaaagttcaaggttctgggaaatacagaccgccacagtatcaaaaaaatcttataggggctcttttatttgacagtggagagacagcggaagcgactcgtctcaaacggacagctcgtagaaggtatacacgtcggaagaatcgtatttgttatctacaggagattttttcaaatgagatggcgaaagtagatgatagtttctttcatcgacttgaagagtcttttttggtggaagaagacaagaagcatgaacgtcatcctatttttggaaatatagtagatgaagttgcttatcatgagaaatatccaactatctatcatctgcgaaaaaaattggtagattctactgataaagcggatttgcgcttaatctatttggccttagcgcatatgattaagtttcgtggtcattttttgattgagggagatttaaatcctgataatagtgatgtggacaaactatttatccagttggtacaaacctacaatcaattatttgaagaaaaccctattaacgcaagtggagtagatgctaaagcgattctttctgcacgattgagtaaatcaagacgattagaaaatctcattgctcagctccccggtgagaagaaaaatggcttatttgggaatctcattgctttgtcattgggtttgacccctaattttaaatcaaattttgatttggcagaagatgctaaattacagctttcaaaagatacttacgatgatgatttagataatttattggcgcaaattggagatcaatatgctgatttgtttttggcagctaagaatttatcagatgctattttactttcagatatcctaagagtaaatactgaaataactaaggctcccctatcagcttcaatgattaaacgctacgatgaacatcatcaagacttgactcttttaaaagctttagttcgacaacaacttccagaaaagtataaagaaatcttttttgatcaatcaaaaaacggatatgcaggttatattgatgggggagctagccaagaagaattttataaatttatcaaaccaattttagaaaaaatggatggtactgaggaattattggtgaaactaaatcgtgaagatttgctgcgcaagcaacggacctttgacaacggctctattccccatcaaattcacttgggtgagctgcatgctattttgagaagacaagaagacttttatccatttttaaaagacaatcgtgagaagattgaaaaaatcttgacttttcgaattccttattatgttggtccattggcgcgtggcaatagtcgttttgcatggatgactcggaagtctgaagaaacaattaccccatggaattttgaagaagttgtcgataaaggtgcttcagctcaatcatttattgaacgcatgacaaactttgataaaaatcttccaaatgaaaaagtactaccaaaacatagtttgctttatgagtattttacggtttataacgaattgacaaaggtcaaatatgttactgaaggaatgcgaaaaccagcatttctttcaggtgaacagaagaaagccattgttgatttactcttcaaaacaaatcgaaaagtaaccgttaagcaattaaaagaagattatttcaaaaaaatagaatgttttgatagtgttgaaatttcaggagttgaagatagatttaatgcttcattaggtacctaccatgatttgctaaaaattattaaagataaagatttttggataatgaagaaaatgaagatatcttagaggatattgtttta

		acattgaccttatttgaagatagggagatgattgaggaaagacttaaaacatatgctcacctctttgat<br>gataaggtgatgaaacagcttaaacgtcgccgttatactggttggggacgtttgtctcgaaaattgatt<br>aatggtattagggataagcaatctggcaaaacaatattagatttttttgaaatcagatggttttgccaat<br>cgcaattttatgcagctgatccatgatgatagtttgacatttaaagaagacattcaaaaagcacaagtg<br>tctggacaaggcgatagtttacatgaacatattgcaaatttagctggtagccctgctattaaaaaaggt<br>attttacagactgtaaaagttgttgatgaattggtcaaagtaatggggcggcataagccagaaaatatc<br>gttattgaaatggcacgtgaaaatcagacaactcaaaagggccagaaaaattcgcgagagcgtatgaaa<br>cgaatcgaagaaggtatcaaagaattaggaagtcagattcttaaagagcatcctgttgaaaatactcaa<br>ttgcaaaatgaaaagctctatctctattatctccaaaatggaagagacatgtatgtggaccaagaatta<br>gatattaatcgtttaagtgattatgatgtcgatgccattgttccacaaagtttccttaaagacgattca<br>atagacaataaggtcttaacgcgttctgataaaaatcgtggtaaatcggataacgttccaagtgaagaa<br>gtagtcaaaaagatgaaaaactattggagacaacttctaaacgccaagttaatcactcaacgtaagttt<br>gataatttaacgaaagctgaacgtggaggtttgagtgaacttgataaagctggttttatcaaacgccaa<br>ttggttgaaactcgccaaatcactaagcatgtggcacaaattttggatagtcgcatgaatactaaatac<br>gatgaaaatgataaacttattcgagaggttaaagtgattaccttaaaatctaaattagtttctgacttc<br>cgaaaagatttccaattctataaagtacgtgagattaacaattaccatcatgcccatgatgcgtatcta<br>aatgccgtcgttggaactgctttgattaagaaatatccaaaacttgaatcggagtttgtctatggtgat<br>tataaagtttatgatgttcgtaaaatgattgctaagtctgagcaagaaataggcaaagcaaccgcaaaa<br>tatttcttttactctaatatcatgaacttcttcaaaacagaaattacacttgcaaatggagagattcgc<br>aaacgccctctaatcgaaactaatggggaaactggagaaattgtctgggataaagggcgagattttgcc<br>acagtgcgcaaagtattgtccatgccccaagtcaatattgtcaagaaaacagaagtacagacaggcgga<br>ttctccaaggagtcaattttaccaaaaagaaattcggacaagcttattgctcgtaaaaaagactgggat<br>ccaaaaaaatatggtggttttgatagtccaacggtagcttattcagtcctagtggttgctaaggtggaa<br>aaagggaaatcgaagaagttaaaatccgttaaagagttactagggatcacaattatggaaagaagttcc<br>tttgaaaaaaatccgattgacttttagaagctaaaggatataaggaagttaaaaaagacttaatcatt<br>aaactacctaaatatagtctttttgagttagaaaacggtcgtaaacggatgctggctagtgccggagaa<br>ttacaaaaaggaaatgagctggctctgccaagcaaatatgtgaatttttatatttagctagtcattat<br>gaaaagttgaagggtagtccagaagataacgaacaaaaacaattgtttgtggagcagcataagcattat<br>ttagatgagattattgagcaaatcagtgaattttctaagcgtgttattttagcagatgccaatttagat<br>aaagttcttagtgcatataacaaacatagagacaaaccaatacgtgaacaagcagaaaatattattcat<br>ttatttacgttgacgaatcttggagctcccgctgcttttaaatattttgatacaacaattgatcgtaaa<br>cgatatacgtctacaaaagaagttttagatgccactcttatccatcaatccatcactggtctttatgaa<br>acacgcattgatttgagtcagctaggaggtgactaa
mrfp1	gene[7]	atggcttcctccgaagacgttatcaaagagttcatgcgtttcaaagttcgtatggaaggttccgttaac<br>ggtcacgagttcgaaatcgaaggtgaaggtgaaggtcgtccgtacgaaggtacccagaccgctaaactg<br>aaagttaccaaaggtggtccgctgccgttcgcttgggacatcctgtccccgcagttccagtacggttcc<br>aaagcttacgttaaacacccggctgacatcccggactacctgaaactgtccttcccggaaggtttcaaa<br>tgggaacgtgttatgaacttcgaagacggtggtgttgttaccgttacccaggactcctccctgcaagac<br>ggtgagttcatctacaaagttaaactgcgtggtaccaacttcccgtccgacggtccggttatgcagaaa<br>aaaaccatgggttgggaagcttccaccgaacgtatgtacccggaagacggtgctctgaaaggtgaaatc<br>aaaatgcgtctgaaactgaaagacggtggtcactacgacgctgaagttaaaaccacctacatggctaaa<br>aaaccggttcagctgccgggtgcttacaaaaccgacatcaaactggacatcacctcccacaacgaagac<br>tacaccatcgttgaacagtacgaacgtgctgaaggtcgtcactccaccggtgcttaataa
yfp	gene[8]	atggtgagcaagggcgaggagctgttcaccggggtggtgcccatcctggtcgagctggacggcgacgta<br>aacggccacaagttcagcgtgtccggcgagggcgagggcgatgccacctacggcaagctgaccctgaag<br>ttcatctgcaccaccggcaagctgcccgtgccctggcccaccctcgtgaccaccttcggctacggcctg<br>caatgcttcgcccgctaccccgaccacatgaagctgcacgacttcttcaagtccgccatgcccgaaggc<br>tacgtccaggagcgcaccatcttcttcaaggacgacggcaactacaagacccgcgccgaggtgaagttc<br>gagggcgacaccctggtgaaccgcatcgagctgaagggcatcgacttcaaggaggacggcaacatcctg<br>gggcacaagctggagtacaactacaacagccacaacgtctatatcatggccgacaagcagaagaacggc<br>atcaaggtgaacttcaagatccgccacaacatcgaggacggcagcgtgcagctcgccgaccactaccag<br>cagaacacccccatcggcgacggccccgtgctgctgcccgacaaccactacctgagctaccagtccgcc<br>ctgagcaaagaccccaacgagaagcgcgatcacatggtcctgctggagttcgtgaccgccgccgggatc<br>actctcggcatggacgagctgtacaagtaataa
araC	gene[3]	atggctgaagcgcaaaatgatcccctgctgccgggatactcgtttaatgcccatctggtggcgggttta<br>acgccgattgaggccaacggttatctcgatttttttatcgaccgaccgctgggaatgaaaggttatatt<br>ctcaatctcaccattcgcggtcagggggtggtgaaaaatcagggacgagaatttgtttgccgaccgggt<br>gatattttgctgttcccgccaggagagattcatcactacggtcgtcatccggaggctcgcgaatggtat<br>caccagtgggtttactttcgtccgcgcgcctactggcatgaatggcttaactggccgtcaatatttgcc<br>aatacggggttctttcgcccggatgaagcgcaccagccgcatttcagcgacctgtttgggcaaatcatt<br>aacgccgggcaaggggaagggcgctattcggagctgctggcgataaatctgcttgagcaattgttactg<br>cggcgcatggaagcgattaacgagtcgctccatccaccgatggataatcgggtacgcgaggcttgtcag<br>tacatcagcgatcacctggcagacagcaattttgatatcgccagcgtcgcacagcatgtttgcttgtcg<br>ccgtcgcgtctgtcacatcttttccgccagcagttagggattagcgtcttaagctggcgcgaggaccaa<br>cgtatcagccaggcgaagctgcttttgagcaccacccggatgcctatcgccaccgtcggtcgcaatgtt<br>ggttttgacgatcaactctatttctcgcgggtatttaaaaaatgcaccggggccagcccgagcgagttc<br>cgtgccggttgtgaagaaaaagtgaatgatgtagccgtcaagttgtcataa
lacI	gene[2]	atgaaaccagtaacgttatacgatgtcgcagagtatgccggtgtctcttatcagaccgtttcccgcgtg<br>gtgaaccaggccagccacgtttctgcgaaaacgcgggaaaaagtggaagcggcgatggcggagctgaat<br>tacattcccaaccgcgtggcacaacaactggcgggcaaacagtcgttgctgattggcgttgccacctcc<br>agtctggccctgcacgcgccgtcgcaaattgtcgcggcgattaaatctcgcgccgatcaactgggtgcc<br>agcgtggtggtgtcgatggtagaacgaagcggcgtcgaagcctgtaaagcggcggtgcacaatcttctc<br>gcgcaacgcgtcagtgggctgatcattaactatccgctggatgaccaggatgccattgctgtggaagct<br>gcctgcactaatgttccggcgttatttcttgatgtctctgaccagacacccatcaacagtattattttc<br>tcccatgaggacggtacgcgactgggcgtggagcatctggtcgcattgggtcaccagcaaatcgcgctg<br>ttagcgggcccattaagttctgtctcggcgcgtctgcgtctggctggctggcataaatatctcactcgc<br>aatcaaattcagccgatagcggaacgggaaggcgactggagtgccatgtccggttttcaacaaaccatg<br>caaatgctgaatgagggcatcgttcccactgcgatgctggttgccaacgatcagatggcgctgggcgca<br>atgcgcgccattaccgagtccgggctgcgcgttggtgcggatatctcggtagtgggatacgacgatacc<br>gaagatagctcatgttatatcccgccgttaaccaccatcaaacaggattttcgcctgctggggcaaacc<br>agcgtggaccgcttgctgcaactctctcagggccaggcggtgaagggcaatcagctgttgccagtctca

		ctggtgaaaagaaaaaccaccctggcgcccaatacgcaaaccgcctctccccgcgcgttggccgattca<br>ttaatgcagctggcacgacaggtttcccgactggaaagcgggcagtgataa
tetR	gene[2]	atgtccagattagataaaagtaaagtgattaacagcgcattagagctgcttaatgaggtcggaatcgaa<br>ggtttaacaacccgtaaactcgcccagaagctaggtgtagagcagcctacattgtattggcatgtaaaa<br>aataagcgggctttgctcgacgccttagccattgagatgttagataggcaccatactcacttttgccct<br>ttagaaggggaaagctggcaagattttttacgtaataacgctaaaagttttagatgtgctttactaagt<br>catcgcgatggagcaaaagtacatttaggtacacggcctacagaaaaacagtatgaaactctcgaaaat<br>caattagcctttttatgccaacaaggtttttcactagagaatgcattatatgcactcagcgctgtgggg<br>cattttactttaggttgcgtattggaagatcaagagcatcaagtcgctaaagaagaaagggaaacacct<br>actactgatagtatgccgccattattacgacaagctatcgaattatttgatcaccaaggtgcagagcca<br>gccttcttattcggccttgaattgatcatatgcggattagaaaaacaacttaaatgtgaaagtgggtcc<br>taa
phlF	gene[2]	atggcacgtaccccgagccgtagcagcattggtagcctgcgtagtccgcatacccataaagcaattctg<br>accagcaccattgaaatcctgaaagaatgtggttatagcggtctgagcattgaaagcgttgcacgtcgt<br>gccggtgcaagcaaaccgaccatttatcgttggtggaccaataaagcagcactgattgccgaagtgtat<br>gaaaatgaaagcgaacaggtgcgtaaatttccggatctgggtagctttaaagccgatctggattttctg<br>ctgcgtaatctgtggaaagtttggcgtgaaaccatttgtggtgaagcatttcgttgtgttattgcagaa<br>gcacagctggaccctgcaaccctgacccagctgaaagatcagtttatggaacgtcgtcgtgagatgccg<br>aaaaaactggttgaaaatgccattagcaatggtgaactgccgaaagataccaatcgtgaactgctgctg<br>gatatgatttttggtttttgttggtatcgcctgctgaccgaacagctgaccgttgaacaggatattgaa<br>gaatttaccttcctgctgattaatggtgtttgtccgggtacacagcgttaa
TrrnB	terminator[5]	gaagcttgggcccgaacaaaaactcatctcagaagaggatctgaatagcgccgtcgaccatcatcatca<br>tcatcattgagtttaaacggtctccagcttggctgttttggcggatgagagaagattttcagcctgata<br>cagattaaatcagaacgcagaagcggtctgataaaacagaatttgcctggcggcagtagcgcggtggtc<br>ccacctgaccccatgccgaactcagaagtgaaacgccgtagcgccgatggtagtgtggggtctccccat<br>gcgagagtagggaactgccaggcatcaaataaaacgaaaggctcagtcgaaagactgggcctttcgttt<br>tatctgttgtttgtcggtgaact
BBa_B0015	terminator	ccaggcatcaaataaaacgaaaggctcagtcgaaagactgggcctttcgttttatctgttgtttgtcgg<br>tgaacgctctctactagagtcacactggctcaccttcgggtgggcctttctgcgtttata
ECK120029600	terminator[9]	ttgagaagagaaaagaaaaccgccgatcctgtccaccgcattactgcaaggtagtggacaagaccggcg<br>gtcttaagtttttggctgaa
ECK120033737	terminator[9]	ggaaacacagaaaaaagcccgcacctgacagtgcgggcttttttttcgaccaaagg
L3S3P21	terminator[9]	ccaattattgaaggcctccctaacgggggcctttttttgtttctggtctccc
L3S2P55	terminator[9]	ctcggtaccaaagacgaacaataagacgctgaaaagcgtcttttttcgttttggtcc
L3S2P21	terminator[9]	ctcggtaccaaattccagaaaagaggcctcccgaaaggggggccttttttcgttttggtcc
L3S2P11	terminator[9]	ctcggtaccaaattccagaaaagagacgctttcgagcgtcttttttcgttttggtcc

VIII. Supplementary References

1. Anderson, J. C., Voigt, C. A. & Arkin, A. P. Environmental signal integration by a modular AND gate. *Mol. Syst. Biol.* **3,** (2007).
2. Stanton, B. C. *et al.* Genomic mining of prokaryotic repressors for orthogonal logic gates. *Nat. Chem. Biol.* **10,** 99–105 (2014).
3. Moon, T. S. *et al.* Construction of a Genetic Multiplexer to Toggle between Chemosensory Pathways in Escherichia coli. *J. Mol. Biol.* **406,** 215–227 (2011).
4. Lou, C., Stanton, B., Chen, Y.-J., Munsky, B. & Voigt, C. A. Ribozyme-based insulator parts buffer synthetic circuits from genetic context. *Nat. Biotechnol.* **30,** 1137–1142 (2012).
5. Qi, L. S. *et al.* Repurposing CRISPR as an RNA-Guided Platform for Sequence-Specific Control of Gene Expression. *Cell* **152,** 1173–1183 (2013).
6. Jinek, M. *et al.* A Programmable Dual-RNA–Guided DNA Endonuclease in Adaptive Bacterial Immunity. *Science* **337,** 816–821 (2012).
7. Campbell, R. E. *et al.* A monomeric red fluorescent protein. *Proc. Natl. Acad. Sci.* **99,** 7877–7882 (2002).
8. Cormack, B. P., Valdivia, R. H. & Falkow, S. FACS-optimized mutants of the green fluorescent protein (GFP). *Gene* **173,** 33–38 (1996).
9. Chen, Y.-J. *et al.* Characterization of 582 natural and synthetic terminators and quantification of their design constraints. *Nat. Methods* **10,** 659–664 (2013).
